# Causes de décès des patients infectés par le VIH dans le Centre tunisien

**DOI:** 10.11604/pamj.2016.25.105.9748

**Published:** 2016-10-21

**Authors:** Jihène Chelli, Foued Bellazreg, Abir Aouem, Zouhour Hattab, Hèla Mesmia, Nadia Ben Lasfar, Wissem Hachfi, Tasnim Masmoudi, Mohamed Chakroun, Amel Letaief

**Affiliations:** 1 Service de Maladies Infectieuses, CHU Farhat Hached, Sousse 4000, Tunisie; 2 Service de Maladies Infectieuses, CHU Fattouma Bourguiba, Monastir 5000, Tunisie; 3 Service de Médecine Légale, CHU Farhat Hached, Sousse 4000, Tunisie

**Keywords:** Causes de décès, facteurs de risque, infections opportunistes, Tunisie, VIH, Causes of death, risk factors, opportunistic infections, Tunisia, HIV

## Abstract

La trithérapie antirétrovirale a contribué à une baisse considérable de la mortalité liée au VIH. Les causes de décès sont dominées par les infections opportunistes dans les pays en voie de développement et par les maladies cardiovasculaires et les cancers dans les pays développés. L’objectif était de déterminer les causes et les facteurs de risque de décès des patients infectés par le VIH dans le Centre Tunisien. Une étude transversale auprès des patients infectés par le VIH âgés de plus de 15 ans suivis à Sousse et à Monastir entre 2000 et 2014. Le décès était considéré lié au VIH si la cause était un évènement classant SIDA ou s’il était la conséquence d’une infection opportuniste d’étiologie indéterminée avec des CD4 < 50/mm^3^, non lié au VIH si la cause n’était pas un évènement classant SIDA, et de cause inconnue si aucune information n’était disponible. Deux cents treize patients, 130 hommes (61%) et 83 femmes (39%), d’âge moyen 40±11 ans ont été inclus. Cinquante quatre patients sont décédés, avec une mortalité de 5,4/100 patients-années. La mortalité annuelle a baissé de 5,8% en 2000-2003 à 2,3% en 2012-2014. La survie était de 72% à 5 ans et de 67% à 10 ans. Les décès étaient liés au VIH dans 70,4% des cas. Les causes de décès les plus fréquentes étaient la pneumocystose pulmonaire et la cryptococcose neuroméningée dans 6 cas (11%) chacune. Les facteurs de risque de décès étaient les antécédents d’infections opportunistes, la durée de la trithérapie antirétrovirale < 12 mois et le tabagisme. Le renforcement du dépistage, l’initiation précoce de la trithérapie antirétrovirale, et la lutte contre le tabagisme sont nécessaires afin de réduire la mortalité chez les patients infectés par le VIH en Tunisie.

## Abstract (en)

Antiretroviral tritherapy has contributed to a considerable reduction in HIV-related mortality. The causes of death are dominated by opportunistic infections in developing countries and by cardiovascular diseases and cancer in developed countries. To determine the causes and risk factors associated with death in HIV-infected patients in two Tunisian medical centers. cross-sectional study of HIV-infected patients over 15 years treated at Sousse and Monastir medical centers between 2000 and 2014. Death was considered related to HIV if its primary cause was AIDS-defining illness or if it was due to an opportunistic infection of unknown etiology with CD4 < 50 cells/mm^3^; it was considered unrelated to HIV if its primary cause wasn’t an AIDS defining illness or if it was due to an unknown cause if no information was available. Two hundred thirteen patients, 130 men (61%) and 83 women (39%), average age 40 ± 11 years were enrolled in the study. Fifty four patients died, the mortality rate was 5.4/100 patients/year. Annual mortality rate decreased from 5.8% in 2000-2003 to 2.3% in 2012-2014. Survival was 72% at 5 years and 67% at 10 years. Death events were associated with HIV in 70.4% of cases. The leading causes of death were pneumocystis carinii pneumonia and cryptococcal meningitis in 6 cases (11%) each. Mortality risk factors were a personal history of opportunistic infections, duration of antiretroviral therapy < 12 months and smoking. Strengthening screening, early initiation of antiretroviral therapy and fight against tobacco are needed to reduce mortality in patients infected with HIV in Tunisia.

## Introduction

La trithérapie antirétrovirale (TAR) a permis une baisse importante de la mortalité globale chez les patients infectés par le virus d’immunodéficience humaine (VIH). En Tunisie, entre décembre 1985, date de la première notification de l’infection par le VIH, et fin 2014, 2008 cas ont été enregistrés, dont 585 cas de décès [1]. La TAR, disponible depuis 2000 dans notre pays, a permis une baisse de la mortalité de 45,1% à 7,8% [2]. Les causes de décès des patients infectés par le VIH sont dominées par les infections opportunistes (IO) dans les pays en voie de développement et par les maladies cardiovasculaires, les hépatites virales et les cancers dans les pays industrialisés [3, 4]. L’objectif de ce travail était de déterminer les causes et les facteurs de risque (FDR) de décès des patients infectés par le VIH dans le Centre Tunisien à l’ère de la TAR.

## Méthodes

**Type de l’étude:** Il s’agit d’une étude transversale des cas d’infection par le VIH suivis dans les services de Maladies Infectieuses de Sousse et de Monastir au Centre Tunisien, entre janvier 2000 et août 2014.

**Population de l’étude:** Nous avons inclus tous les patients âgés de plus de 15 ans au moment de l’étude, ayant une infection par le VIH confirmée par Western-Blot, hospitalisés et/ou suivis à la consultation. Nous avons exclu les patients perdus de vue en cas d’absence d’information sur leurs états de santé depuis plus d’un an. Les caractéristiques cliniques et immuno-virologiques ont été recueillies par un même médecin à partir des dossiers médicaux.

**Causes de décès:** Le taux de mortalité global a été calculé en divisant le nombre de décès par le nombre de patients-années, et le taux de mortalité annuel a été calculé en divisant le nombre de patients décédés pendant une année par le nombre de patients suivis pendant la même année. La cause de décès était définie par la pathologie ou l’accident ayant directement déclenché l’évolution vers le décès. Les causes de décès ont été obtenues à partir des dossiers médicaux pour les patients décédés à l’hôpital, et à partir des membres de la famille ou des amis ou partenaires pour les patients décédés hors de l’hôpital. Dans tous les cas, au moins 3 médecins du service prenant en charge le patient étaient d’accord pour retenir la cause de décès. Le décès était considéré lié au VIH si la cause était un évènement classant SIDA selon la classification du Center for Diseases Control and Prevention (CDC) de 1993 ou s’il était la conséquence d’une IO d’étiologie indéterminée avec des CD4 < 50/mm^3^. Le décès était considéré non lié au VIH si la cause de décès n’était pas un évènement classant SIDA selon la classification du CDC 1993, et de cause inconnue si aucune information n’était disponible. Nous avons comparé les taux de mortalité avant et après 2010 puisque, dans notre pays, le seuil des CD4 à partir duquel une TAR était indiquée est passé de 200/mm^3^ avant 2010 à 350/mm^3^ à partir de 2010.

**Facteurs de risque de décès:** Les FDR de décès étaient recherchés par une analyse univariée comparant les personnes décédées et les personnes non décédées en utilisant le test de Chi 2 de Pearson et le test exact de Fisher pour les variables catégorielles, et le test T de Student pour les variables continues. Les variables pour lesquelles il y avait une différence statistiquement significative (seuil de signification p<0,05) et celles dont p était < 0,2 ont été introduites dans le modèle de régression logistique de Hosmer et Lemshow afin d’identifier les FDR indépendants de décès. L’odds ratio (OR) et l’intervalle de confiance à 95% (IC 95%) ont été calculés afin d’estimer l’impact du FDR. Les FDR de décès suivants ont été analysés: genre, âge au moment du décès, ancienneté de l’infection par le VIH, nadir des CD4, charge virale plasmatique (CVP), antécédent(s) d’IO, stade C de la classification du CDC, consommation de tabac; consommation d’alcool, utilisation d’une TAR, durée de la TAR. L’analyse des données de survie était réalisée par la courbe d’estimation de survie de Kaplan-Meier.

## Résultats

**Figure 1:**
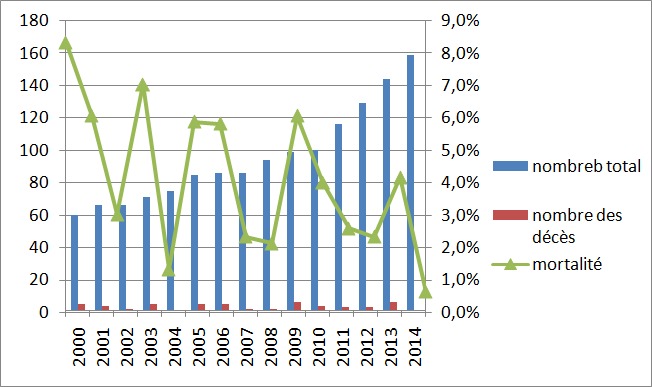
Evolution annuelle du taux de mortalité des patients infectés par le VIH

**Caractéristiques générales des patients - taux de mortalité - survie:** Durant la période d’étude, 260 patients étaient suivis pour une infection par le VIH dont 47 étaient perdus de vue et 213 avaient un suivi régulier. Ces derniers étaient inclus dans l’étude. Il s’agissait de 130 hommes (61%) et 83 femmes (39%) âgés en moyenne de 40±11 ans (18-81 ans). Deux cent deux patients (94,8%) étaient de nationalité tunisienne. Le mode de transmission était sexuel dans 203 cas (95,3%). La durée médiane du suivi était de 3 ans. Cinquante quatre patients, 35 hommes et 19 femmes, âgés en moyenne de 44 ± 11 ans, sont décédés. Le taux de mortalité global était de 5,4/100 patients-années. Le taux de mortalité annuel était de 4,1%. Il avait baissé de 5,8% durant la période 2000-2003 à 2,3% durant la période 2012-2014 (**Figure 1**). Le taux de mortalité était de 4,8% avant 2010 et de 2,7% après. La survie était de 72% à 5 ans et de 67% à 10 ans. Chez les patients non décédés suite à une IO grave révélatrice de l’infection par le VIH (n=201), la survie était de 77% à 5 ans et de 70% à 10 ans. Chez les 54 patients décédés, l’infection par le VIH était diagnostiquée depuis une durée médiane de 50 mois et le nadir des CD4 était < 50/mm^3^ dans 25 cas (46%). Des antécédents d’IO, une consommation de tabac et une consommation d’alcool étaient notés chez respectivement 40/51(78%), 23/40 (57%), et 20/39 (51%) des patients. Au moment du décès, 37 patients/46 (80%) étaient sous TAR, les CD4 étaient < 50/mm^3^ dans 25 cas/32 (78%) et la CVP était >10 000 copies/ml dans 28 cas/30 (93%).

**Causes de décès:** Les décès étaient liés au VIH dans 38 cas (70,4%), non liés au VIH dans 8 cas (14,8%), et de cause inconnue dans 8 cas (14,8%) (Tableau 1). Le décès était survenu suite à une IO révélatrice de l’infection par le VIH dans 12 cas (22,2%), à cause d’une IO ou d’un cancer lié au VIH survenant au cours du suivi dans 13 cas (24%) et 4cas (7,4%) respectivement, et de cause indéterminée dans 9 cas (16,7%). Les causes de décès les plus fréquentes étaient la pneumocystose pulmonaire et la cryptococcose neuroméningée dans 6 cas (11,1%) chacune, le lymphome dans 4 cas (7,4%) et la toxoplasmose cérébrale dans 3 cas (5,6%). Les deux principales localisations des IO étaient cérébrales dans 15 cas (27,9%) et pulmonaires dans 8 cas (14,8%). Avant 2010 le décès était lié au VIH dans 26/37 cas (70,2%), non lié au VIH dans 4/37 cas (10,9%) et de cause inconnue dans 7/37 cas (18,9%); après 2010, ces taux étaient de 70,6%, 23,6% et 5,8%, respectivement. La différence n’était pas significative (p=0,4). En analyse univariée, les FDR de décès étaient la durée de l’infection < 12 mois, le taux de CD4 au moment du diagnostic < 200/mm^3^, le nadir CD4 <50/mm^3^, les antécédent(s) d’IO, la durée de la TAR < 12 mois et le tabac. En analyse multivariée, les FDR de décès étaient les antécédents d’IO, la durée de la TAR < 12 mois et le tabac (Tableau 2).

**Tableau 1:** Causes de décès des patientsinfectés par le VIH

Causes de décès	n (%)
**Liées au VIH**	**38 (70,4)**
Infections opportunistes	24 (44,4)
Pneumocystose	6 (11,1)
Cryptococcose	6 (11,1)
Toxoplasmose	3 (5,6)
Autres+	9 (16,7)
Cancers	5 (9,2)
Lymphome	4 (7,4)
Sarcome de kaposi	1 (1,8)
Indéterminée	9 (16,7)
**Non liées au VIH++**	**8 (14,8)**
**Inconnues**	**8 (14,8)**
Total	54 (100)

+ Tuberculose (2), leucoencéphalite multifocale progressive (2), infection à CMV (2); encéphalite VIH (2), cryptosporidiose (1).

++ Pneumopathie grave (3), pneumopathie à Scedosporuim (1), méningo-encéphalite à pneumocoque (1), cancer du larynx (1), hépatite virale C (1), déshydratation aigue (1)

**Tableau 2:** Caractéristiques cliniques et immuno-virologiques des patients et facteurs de risque de décès

	Analyse univariée				Analyse multivariée	
	Décédés (n, %)	Non décédés (n, %)	p	OR (IC 95%)	p	OR (IC 95%)
Genre			0,5	0,8 [0,42-1,53]		
Féminin	19 (35,2)	64 (40,3)				
Masculin	35 (64,8)	95 (59,7)				
Age (années)			0,05	1,87 [0,99-3,5]		
> 40	29 (55,8)	64 (40,3)				
< 40	23 (44,2)	95 (59,7)				
ancienneté del’infection par le VIH			0,001	4,9 [2,24-10,96]		
<12 mois	22 (57,9)	23 (21,7)				
>12 mois	16 (42,1)	83 (78,3)				
CD4 au moment du diagnostic (/mm^3^)			0,001	6,05 [2,23-16,41]		
< 200	32 (86,6)	74 (51,4)				
> 200	5 (13,5)	70 (48,5)				
CV^1^ VIH au moment du diagnostic (copies/ml)			0,5	0,67 [0,18-2,467]		
< 10 000	3 (12,5)	21 (17,5)				
> 10 000	21 (87,5)	99 (82,5)				
nadir des CD4 (/mm^3^)						
< 50	45 (67,2)	22 (32,8)	0,003	0,23 [0,08-0,66]		
> 50	44 (89,8)	5 (10,2)				
Antécédents d’IO^2^stade C			0,001	11,3 [3,55-36,02]	0,001	11,3 [3,5-36,02]
Oui	41 (80,4)	61 (41,8)				
Non	10 (19,6)	85 (58,2)				
Tabac			0,03	2,18 [1,04-4,5]	0,008	4,3 [1,47-12,36]
Oui	26 (65)	56 (45,9)				
Non	14 (35)	66 (54,1)				
Alcool			0,15	1,7 [0,82-3,52]		
Oui	29 (53,8)	48 (40,6)				
Non	18 (46,1)	70 (64,8)				
Trithérapie antirétrovirale			0,64	0,8 [0,33-1,95]		
Oui	38 (82,6)	135(85,4)				
Non	8 (17,4)	23 (14,6)				
durée de la TAR^3^			0,001	3,82 [1,78-8,17]	0,001	1,08 [1,01-1,04]
< 12 mois	19 (50)	28 (20,7)				
> 12 mois	19 (50)	107(79,3)				

1 charge virale

2 infection opportuniste

3 trithérapie antirétrovirale

## Discussion

Dans la littérature, la mortalité globale varie de 1,27/100 patients-années en Europe, Amérique du Nord et Australie, à 3,2/100 patients-années au Brésil, et 5,19/100 patients-années en Corée [5–7]. Notre étude montre que la mortalité globale des PVVIH était élevée de 5,4/100 patients-années avec une mortalité annuelle qui diminue au cours des 3 dernières années (allant de 6,4% au cours des années 2000-2003 à 2,3% au cours des années 2012-2014). Notre étude montre l’initiation tardive de la TAR chez les patients décédés puisque 46% d’entre eux avaient un nadir de CD4 < 50/mm^3^ et 78% avaient des antécédents d’IO. Ceci explique la fréquence élevée des causes de décès liées au VIH (70,4%) qui n’a pas connu une baisse significative au cours de la période de l’étude. En Tunisie, la pneumocystose pulmonaire, la cryptococcose neuro-méningée et la toxoplasmose sont les IO les plus fréquemment associées au décès. Dans d’autres pays en voie de développement, la tuberculose et la cryptosporidiose apparaissent fréquemment comme des causes de décès associées au VIH [4, 7]. Par contre dans les pays industrialisés les causes les plus fréquentes de décès sont non liées au VIH telles que les maladies hépatiques et les maladies cardiovasculaires [3, 8, 9] (Tableau 3). Notre étude confirme également la fréquence des lymphomes (7,4%) comme cause de décès et la rareté de cancers non liés au VIH, fréquents dans certaines régions [3, 8, 9]. Bien qu’on n’ait pas pu évaluer l’impact de l’observance thérapeutique sur la mortalité des PVVIH, la fréquence de survenue des IO sous antirétroviraux laisse prévoir une mauvaise observance. De même, la prophylaxie au cotrimoxazole chez les PVVIH décédées par pneumocystose et toxoplasmose n’a pu être vérifiée dans tous les cas. Les trois FDR de décès identifiés dans l’analyse multivariée sont les antécédents d’IO, la durée de la TAR < 12 mois et le tabac. L’initiation précoce du TAR avec un suivi régulier et une bonne observance thérapeutique permet d’obtenir une suppression virale durable entrainant une amélioration de la survie avec une meilleure qualité de vie et une réduction de la transmission du virus. Cet objectif peut être atteint d’une part par un meilleur accès aux soins avec une prescription raisonnée d’antirétroviraux et d’autre part par la mise en place d’une stratégie de dépistage permettant la connaissance précoce du statut sérologique de la personne avec une orientation rapide à un service de prise en charge. Le tabagisme associé ainsi que les complications métaboliques de certains antirétroviraux représentent des FDR majeurs des maladies cardiovasculaires responsables d’une mortalité non négligeable dans les pays industrialisés [3, 10, 11]. Dans notre étude, la fréquence du tabagisme chez les patients infectés par le VIH et l’apparition du tabac comme FDR de décès doivent inciter à intégrer dans la prise en charge globale de l’infection par le VIH une stratégie de lutte contre le tabac et de sevrage tabagique afin de réduire la mortalité liée aux maladies cardiovasculaires.

**Tableau 3:** Causes de décès des patients infectés par le VIH dans la littérature, exprimés en pourcentages

Causes de décès	Maroc (n=1243) 1999-2009 [4]	Corée (n=327) 1998-2006 [7]	Brésil (n=1538) 1997-2006 [5]	Europe, Am. du Nord, Australie (n=49731) 1999-2011 [3]	France (n=82000) (2010) [8, 9]	Notre étude (n=213) 2000-2014
**Liées au VIH**	**96**	**55,9**	**49,1**	**29**	**25**	**70,4**
**IO^1^**	**85**	**50**	**37,6**	**-**	**-**	**44,4**
Tuberculose	35	22,1	-	-	4	3,7
Pneumocystose	0	10,3	-	-	-	11,1
Toxoplasmose	9	0	-	-	-	5,6
Cryptococcose	13	1,5	-	-	-	11,1
Cryptosporidiose	19	0	-	-	-	1,8
Mycob.^2^ atypique	2	0	-	-	-	0
Syndrome cachectique	1	10,3	-	-	-	0
SIRI^3^	6	0	-	-	-	0
Infection à CMV	0	1,5	-	-	-	3,7
LEMP^4^	0	1,5	-	-	3	3,7
Encéphalite à VIH	0	3	-	-	-	3,7
**Cancers**	**11**	**5,9**	**7,5**	**-**	**9,3**	**9,2**
Lymphomes NH^5^	4	5,9	-	-	7,3	7,4
Sarcome de Kaposi	6	0	-	-	1,5	1,8
Cancer du col	1	0	-	-	0,5	0
**Indéterminée**	**0**	**4,4**	**4**	**-**	**-**	**16,7**
**Non liées au VIH**	**4**	**36,8**	**43,4**	**64**	**67**	**14,8**
Aspergillose	1	0	-	-	-	0
Maladie hépatique	2	7,4	3,5	13	11	1,8
Cancers	1	4,4	3,5	15	27	1,8
Infection bactérienne	0	5,9	8,4	7	-	7,4
MCV^6^	0	7,4	4	11	9	0
Maladie gastro-intestinale	0	2,9	-	-	10	0
Accident	0	1,5	-	2	-	0
Suicide	0	7,4	-	4	-	0
Homicide	0	0	-	1	5	0
overdose	0	0	-	3	-	0
Insuffisance rénale	0	0	-	1	-	0
Pancréatite	0	0	0	0	-	0
Autres	0	0	23,9	6	5	3,7
**Inconnue**	**0**	**2,9**	**7,5**	**7**	**8**	**14,8**
**Total décès**	**91**	**68**	**226**	**3909**	**728**	**54**

1 infections opportunistes

2 mycobactériose

3 syndrome inflammatoire de restauration immunitaire

4 leucoencéphalite multifocale progressive

5 non Hodgkinien

6 maladie cardiovasculaire.

## Conclusion

Notre étude montre que les décès des patients infectés par le VIH étaient fréquemment liés au VIH en raison de l’initiation tardive des antirétroviraux. Le tabac apparaît comme autre FDR de décès chez ces patients. Le renforcement du dépistage, l’initiation précoce de la TAR, le renforcement de l’arsenal thérapeutique des antirétroviraux, et la lutte contre le tabagisme sont les principales composantes à prendre en considération dans le but de réduire la mortalité chez les patients infectés par le VIH en Tunisie.

### Etat des connaissances actuelles sur le sujet

- La trithérapie antirétrovirale a contribué à une baisse considérable de la mortalité liée au VIH;
- Les causes de décès sont dominées par les infections opportunistes dans les pays en voie de développement et par les maladies cardiovasculaires et les cancers dans les pays développés.

### Contribution de notre étude à la connaissance

- Dans le Centre tunisien, à l’ère de la trithérapie antirétrovirale la mortalité des patients infectés par le VIH est de 5,4/100 patients-années;
- Les décès sont liés au VIH dans 70,4% des cas et les causes de décès les plus fréquentes sont la pneumocystose pulmonaire et la cryptococcose neuroméningée;
- Les facteurs de risque de décès sont les antécédents d’infections opportunistes, la durée de la trithérapie antirétrovirale < 12 mois et le tabagisme.
